# The Effects of Taoren-Honghua Herb Pair on Pathological Microvessel and Angiogenesis-Associated Signaling Pathway in Mice Model of CCl_4_-Induced Chronic Liver Disease

**DOI:** 10.1155/2016/2974256

**Published:** 2016-05-11

**Authors:** Shengyan Xi, Lifeng Yue, Mengmeng Shi, Ying Peng, Yangxinzi Xu, Xinrong Wang, Qian Li, Zhijun Kang, Hanjing Li, Yanhui Wang

**Affiliations:** ^1^Department of Traditional Chinese Medicine, Medical College of Xiamen University, Xiamen, Fujian 361102, China; ^2^Dongzhimen Hospital, Beijing University of Chinese Medicine, Beijing 100700, China; ^3^Department of Internal Medicine, University of Manitoba, Winnipeg, MB, Canada R3E 3P4; ^4^School of Basic Medical Science, Beijing University of Chinese Medicine, Beijing 100029, China; ^5^West China Medical Center, Sichuan University, Chengdu, Sichuan 610041, China

## Abstract

Chronic liver disease is one of the most common diseases that threaten human health. Effective treatment is still lacking in western medicine.* Semen Persicae* (Taoren) and* Flos Carthami* (Honghua) are known to relieve acute hepatic injury and inflammation, improve microcirculation, and reduce tissue fiber. The aim of our study is to investigate the potential mechanisms of Taoren-Honghua Herb Pair (THHP) in murine model of chronic liver disease caused by Carbon Tetrachloride (CCl_4_). Mice were randomly divided into seven groups: (1) blank, (2) model, (3) control (colchicine, 0.1 mg/kg), (4) THHP (5.53, 2.67, and 1.33 g/kg), and (5) Tao Hong Siwu Decoction (THSWD) (8.50 g/kg). Histological change and microvessels density were examined by microscopy. Hepatic function, serum fibrosis related factors, and hepatic vascular endothelial growth factor (VEGF) were measured with ELISA. VEGF, kinase insert domain-containing receptor (KDR), Flt-1, and Akt mRNA expression in hepatic tissue were determined with PCR. Tissues of Akt, pAkt, KDR, and Flt-1 were measured with western blotting. Data from this study showed that THHP improved hepatic function and restrained the hepatic inflammation and fibrosis. Its role in inhibiting pathological angiogenesis and hepatic fibrogenesis may be through affecting the angiogenesis-associated VEGF and its upstream and downstream signaling pathways.

## 1. Introduction

Chronic liver disease describes a wide range of liver complications including fatty liver, hepatitis, fibrosis, cirrhosis, and hepatocellular carcinoma. In particular, hepatic inflammation and fibrosis are hallmarks of the progression. Researches on prevention and treatment of chronic hepatitis and hepatic fibrosis are of major importance. At present, there is no effective treatment in Western medicine that produces minimum adverse effects [1, 2]. Attention has been turned to natural herbal medicines such as Chinese traditional medical formulas, which can offer great opportunities in preventing and treating chronic liver diseases with minimum side effects.

Chinese medical formulas are prepared from a complex combination of natural herbs, minerals, and animal products, which are thus considered to be multifunctional therapeutic agents that exert their function in a holistic way. Clinical use of traditional Chinese medicine for chronic liver disease emphasizes promoting blood circulation and dissolving stasis to relieve symptoms. Taoren-Honghua Herb Pair (THHP) is composed of* Semen Persicae* (Taoren) and* Flos Carthami* (Honghua) with the function of promoting blood circulation. Formulas composed mainly of these two ingredients are used extensively in China for the treatments of chronic hepatitis and hepatic fibrosis and have generated satisfactory therapeutic efficacy [3–7]. Thus, it is beneficial to elucidate the underlining mechanisms in which THHP exerts its function.

In recent years, studies have indicated that, during the development of chronic liver disease, tissues undergo pathological angiogenesis and fibrotic collagen synthesis, which ultimately lead to distortion of normal architecture making recovery hard to achieve [8]. Chronic liver disease with inflammation and hepatic fibrosis may cause the expression increase of vascular endothelial growth factor (VEGF) and its receptor kinase insert domain-containing receptor (KDR) and Flt-1 and phosphorylated serine/threonine kinase B (pAkt) and the activation of the PI3K (phosphoinositide 3-kinase)/Akt signaling pathway promotes the production of VEGF-A in hepatocytes and expression of VEGFR-2 [9–11]. Therefore, selecting these markers to research would clarify the mechanisms of traditional Chinese medicine treating chronic hepatitis and hepatic fibrosis.

This study investigated the effect of THHP on hepatic function and fibrosis through proteins and mRNA expression correlated with pathological angiogenesis from vascular endothelial growth factor signaling pathway in chronic liver disease model mice induced by CCl_4_. The results of the experiment might provide a certain insight as to the possible mechanisms of action of THHP.

## 2. Materials and Methods

### 2.1. Materials

In this study, we used the following materials: Carbon Tetrachloride (CCl_4_), 500 mL per bottle, product lot number 20140102, was purchased from the Dahao Fine Chemical Co., Ltd. (Shantou, China). Van Gieson staining solution, product lot number SBJ-0297, was purchased from Nanjing SenBeiJia Biological Technology Co., Ltd. (Nanjing, China). CSB-E08121m Mice HA ELISA Kit, product lot number G01012078, CSB-E04645m Mice LN ELISA Kit, product lot number G03012041, CSB-E13878m Mice Albumin ELISA Kit, product lot number G04012056, CSB-E16539m Mice ALT/GPT ELISA Kit, product lot number G07011201, CSB-E12649m Mice AST ELISA kit, product lot number G09012089, and CSB-E04756m Mice VEGF ELISA Kit, product lot number G11012036, were purchased from Cusabio Biotech Co., Ltd. (Wuhan, China). CD34 mouse monoclonal antibody (sc-74499), product lot number L7109, Akt polyclonal antibody (sc-5298), product lot number L2102, pAkt polyclonal antibody (sc-135650), product lot number L3950, and KDR polyclonal antibody (sc-6251), product lot number L2450, were purchased from Santa Cruz Biotechnology Co., Ltd. (Dallas, USA). Biotin-Conjugated Goat Anti-Mouse IgG (H+L) (SA00001-1), product lot number A5036, Peroxidase-Conjugated Streptavidin (SA00001-0), product lot number A3017, and Flt-1 polyclonal antibody (13687-1-AP), product lot number A5691, were purchased from Proteintech Group, Inc. (Wuhan, China). Hematoxylin staining solution (C0107), product lot number B131001, was purchased from Beyotime Biotech Co., Ltd. (Shanghai, China). Goat serum (C-0005), product lot number G10719, was purchased from Shanghai Haoran Biotech Co., Ltd. (Shanghai, China). Neutral gum (10004160), product lot number 20140411, methanol (10014108), product lot number 20140705, H_2_O_2_ (S32338102), product lot number 20140321, EDTA (10009617), product lot number 20130907, and SDS, product lot number 30166428, were purchased from Sinopharm Chemical Reagent Co., Ltd. (Shanghai, China). DAB Substrate Kit (34002), product lot number QJ223393, was purchased from Thermo Fisher Scientific Inc. (Waltham, USA). R250 Coomassie brilliant blue, product lot number 29721, was purchased from Aladdin Reagent Co., Ltd. (Shanghai, China). ECL Luminescence Kit, product lot number P004, was purchased from Vigorous Biotechnology Beijing Co., Ltd. (Beijing, China). Reverse transcription-polymerase chain reaction primers were compounded by Sangon Biotech (Shanghai) Co., Ltd. (Shanghai, China) and Trizol Extraction Kit, AMV First Strand cDNA Synthesis Kit (SK2445), Ethidium Bromide, SanPrep Column DNA Gel Extraction Kit (SK8131), SanPrep Column Plasmid DNA Mini-Preps Kit (SK8191), and Quantitive PCR Reagent (ABI SYBR Green PCR Master Mix) (2x) were from Sangon Biotech (Shanghai) Co., Ltd. (Shanghai, China).

### 2.2. Medicinal Preparation

THHP, which is composed of* Semen Persicae* (Taoren) granule (product lot number 1405067) 0.9 g [equal to* Semen Persicae* (Taoren) 9 g] and* Flos Carthami* (Honghua) granule (product lot number 1405019) 2 g [equal to* Flos Carthami* (Honghua) 6 g], was purchased from Jiangyin Tianjiang Pharmaceutical Co., Ltd. (Jiangyin, China). They were directly mixed with distilled water to 0.553 g/mL, 0.267 g/mL, and 0.133 g/mL, respectively. The mixture was preserved in 4°C fridge. THSWD includes* Semen Persicae* (Taoren) 9 g,* Flos Carthami* (Honghua) 6 g,* Radix Rehmanniae* (Shengdi) 12 g,* Radix Paeoniae Rubra* (Baishao) 9 g,* Radix Angelicae Sinensis* (Danggui) 9 g, and* Rhizoma Chuanxiong* (Chuanxiong) 6 g. Total 51 g of crude drug was provided by Xiamen Yanlaifu Pharmaceutical Co., Ltd. (Xiamen, China). These herbs were soaked in water for 20 min and boiled for 30 min to yield 200 mL. The decoction was performed twice to yield final volume of 400 mL. The solution was filtered with carbasus and concentrated to 0.85 g/mL. The remaining decoction was lyophilized into powder and stored at 4°C. Colchicine tablet, product lot number 131126, was purchased from the Xishuangbanna Pharmaceutical Co., Ltd. (Jinghong, China). Colchicine tablet was added to double steamed water to a concentration of 0.01 mg/mL.

### 2.3. Animals

Seventy healthy, 4-week-old, Kunming (KM) mice with specific pathogen-free (SPF) degree, male and female in equal number, weighing 16.0 ± 2.0 g, were provided by the SLAC Laboratory Animal Co. Ltd. in Shanghai, China [License Number SCXK (Hu) 2012-0002]. The mice were bred in animal house (SPF degree) with barrier system assisted with apinoid laminar flow chamber in the Experimental Animal Center of Xiamen University and used for experiment after 1 week.

### 2.4. Experimental Methods

#### 2.4.1. Animal Models, Grouping, and Administration

Seventy mice were divided with random digits table into 7 groups: blank, model, positive control, high, medium, and low THHP dosage, and THSWD group with 10 mice in each. Other than the blank group, mice were injected subcutaneously with 0.1 mL/10 g 40% CCl_4_ and Arachis Oil solution in the left inguen to establish the chronic liver disease model. The injection was administered once every 5 days for 6 weeks with first injection containing double dosage of CCl_4_. The THHP-H, THHP-M, and THHP-L were treated intragastrically with 0.2 mL/10 g body weight of THHP aqueous solution at concentrations of 0.553 g/mL, 0.267 g/mL, and 0.133 g/mL, respectively, once a day, the THSWD group was treated with intragastric administration (0.2 mL/10 g) of 0.85 g/mL THSWD once a day, and the colchicine treated group underwent intragastric administration (0.2 mL/10 g) of 0.01 mg/mL colchicine solution once a day for 6 weeks. Colchicine is a traditionary drug for antagonizing hepatic fibrosis, so it was selected as a positive drug [12, 13]. The model group was given saline solutions by the same method. All treatments began 24 hours after establishing model with CCl_4_ injection at the first time.

#### 2.4.2. Enzyme-Linked Immunosorbent Assay (ELISA) Measurement of Serums AST, ALT, Albumin, HA, and LN

Twenty-four hours after the last administration, 0.8 mL of peripheral blood was collected from each mouse by eyeball extirpation. Blood samples were incubated at room temperature for 60 min, and serums were isolated by centrifuging at 3000 r/min at 4°C for 10 min. After protein extraction solution was added, samples were incubated on iced bath for 30 min and centrifuged at 15000 r/min. Supernatants were collected and stored at −20°C for protein quantification. HA ELISA Kit, LN ELISA Kit, Albumin ELISA Kit, Alanine Aminotransferase (ALT) ELISA Kit, and Aspartate Aminotransferase (AST) ELISA kit were used to assay the concentration of serums HA, LN, Albumin, ALT, and AST, respectively. According to protocol, 100 *μ*L of sample was incubated with 100 *μ*L of VEGF biotin labelled antibody for 60 min at 37°C followed by 3 times' wash. 100 *μ*L of horseradish peroxidase (HRP) labelled avidin fluid was added and incubated for 30 min at 37°C followed by 5 times' wash. Then, 90 *μ*L of substrate solution was added and incubated for 25 min at 37°C. 50 *μ*L of stop solution was added lastly and the optical density (OD) value was read with Multimode Reader at 450 nm wavelength.

#### 2.4.3. VG Staining and Immunohistochemistry Staining

At the time of sacrifice, a small piece of hepatic tissue was collected from each mouse and fixed in 4% formaldehyde solution for 24 h. Paraffin sections (4 *μ*m) were made and stained with Van Gieson (VG) staining solution. All sections were prepared for routine histological evaluations. The hepatic pathological changes were observed by light microscopy at ×100 and morphological changes were recorded. For immunohistochemistry staining microvessels, tissue section was dewaxed and incubated with 3% H_2_O_2_ at room temperature to inactivate endogenous peroxidase. After wash with distilled water and PBS for 5 min, the section was microwaved for 20 mins in 0.01 M citric acid buffer. Tissue was cooled to room temperature and incubated in 20 mL/L goat serum for 10 min followed by incubation at 37°C in CD34 mouse anti-human monoclone antibody (first antibody) and staying overnight at 4°C, with wash with PBS for 5 min thrice. The following day, tissue was incubated in Biotin-Conjugated Goat Anti-Mouse IgG (secondary antibody) for 30 min and washed with PBS. Tissue was then incubated in avidin-biotin complex for 30 min and stained with DAB. Following that, Tissue was stained and differentiated in hematoxylin, followed by dehydration and transparency. Finally, the tissue slide was mounted and observed under the light microscope.

#### 2.4.4. Measuring Standard for Hepatic Microvessel Density (MVD)

The integrated optical density (IOD) of hepatic microvessels was determined by Image-Pro Plus 6.0 image processing system. Three areas with the most abundant capillary vessels, referred to as “hot spots,” were selected at ×100 magnification under light microscope and counted at a magnification of ×400. The average IOD value of the three was set as the hepatic tissue's MVD. Each endotheliocyte or endothelial cell cluster stained brown, which was separated from the adjacent microvessel, hepatic cell, or other connective tissues, was considered as a capillary vessel. In order to avoid disturbance of comparatively large vessels, the vessel walls with thick muscular layer or lumen of blood vessel with more than 8 blood cells were excluded. Each section was inspected and counted by two pathologists separately. If the difference was over 10%, the analysis was repeated.

#### 2.4.5. ELISA Assays of VEGF Protein Content in Hepatic Tissue

At the time of sacrifice, parts of hepatic tissues were washed with demineralized water, cut into fine pieces by ophthalmic scissors, transferred to a homogenizer, exposed to protein extraction solution, incubated on iced bath, and then centrifuged at 15000 r/min. Supernatants were collected and stored at −20°C for protein quantification. Vascular endothelial growth factor (VEGF) ELISA Kit was used to assay the concentration of tissue VEGF according to the above-mentioned protocol.

#### 2.4.6. Real-Time Fluorescent Quantitation PCR Assays of VEGF, Flt-1, KDR, and Akt mRNA Expression in Hepatic Tissues

Total RNAs were extracted from the hepatic tissue using the Trizol Extraction Kit, and then cDNA was synthesized by reverse transcription from 1 *μ*g of total RNA using AMV First Strand cDNA Synthesis Kit. The reverse transcription products were diluted into 100 *μ*L as cDNA templates. Then, the PCR reaction was performed on a LightCycler 480 Software Setup (Amplification System) in a total volume of 20 *μ*L containing 2 *μ*L cDNA templates, 1 *μ*L (10 *μ*m) F-primer, 1 *μ*L (10 *μ*m) R-primer, 10 *μ*L (2×) SYBR Green qPCR Master Mix, and ddH_2_O 6 *μ*L. The primer sequences were as follows: GAPDH, forward 5′-GGTT GTCT CCTG CGAC TTCA-3′, reverse 5′-TGGT CCAG GGTT TCTT ACTC C-3′, and product length 183 bp; VEGF, forward 5′-GTAA CGAT GAAG CCCT GGAG T-3′, reverse 5′-TGTT CTGT CTTT CTTT GGTC TGC-3′, and product length 152 bp; Flt-1, forward 5′-TGAA CGGC TGCC CTAT GAT-3′, reverse 5′-CCGA GCGA TTTG CCTA GTT-3′, and product length 72 bp; KDR, forward 5′-GCAG TCAA GTCC GAAT CCCT-3′, reverse 5′-GAGT TCAT CGCC AACA ATCA T-3′, and product length 116 bp; Akt, forward 5′-TTCT ATGG TGCG GAGA TTGT GT-3′, reverse 5′-CAGC CCGA AGTC CGTT ATCT-3′, and product length 132 bp. The amplification conditions were as follows: predenaturation for 3 min at 95°C, denaturation for 15 s at 95°C, annealing for 20 s at 57°C, and extension for 30 s at 72°C. PCR was performed for 35 cycles. After obtaining the cycle threshold (Ct), the Ct values of the housekeeping genes GAPDH were subtracted from those of target genes to obtain the ΔCt value; ΔCt of different groups was subtracted from ΔCt of normal group to obtain ΔΔCt; and the quantity of mRNA expression of target genes related to internal control genes was presented as 2^−(ΔΔCt)^. And, finally, 5 *μ*L of each PCR product was subjected to electrophoresis on the 2% agarose gel and the ethidium bromide-stained bands were scanned. The experiment was repeated three times.

#### 2.4.7. Western Blot Analysis of Akt, pAkt, Flt-1, and KDR Protein Expression in Hepatic Tissues

Parts of hepatic tissues were cut into small pieces and placed in RIPA buffer lysate to homogenize. The protein concentration was measured using the Coomassie brilliant blue method. Equal concentrations of proteins (100 *μ*g) were separated by 8% SDS-polyacrylamide gel electrophoresis (SDS-PAGE) and electroblotted onto polyvinylidene difluoride membranes. The blots of Akt, pAkt, Flt-1, and KDR were blocked with 1% casein solution for 2 h at room temperature. Blots were incubated for 1 h at 37°C with 1 : 1000 dilution of polyclonal antibody for Akt, pAkt, Flt-1, and KDR diluted with 1% casein solution and rinsed 5 times with PBST buffer for 3 min. Then, blots were incubated with 1 : 10000 dilution of the Peroxidase-Conjugated Streptavidin secondary antibody for 1 h and washed 5 times with PBST buffer. Protein levels were visualized with luminescence solution and X-ray films. Equal lane loading was assessed using *β*-actin. The films were scanned, and the average optical densities of the blots were analyzed with Image-Pro Plus 6.0 image processing system.

#### 2.4.8. Statistical Analysis

The data was presented as mean ± standard deviation. GraphPad Prism 5 software (GraphPad Software Inc., La Jolla, USA) was used to analyze the data. Statistical significance of results of different treatment groups was determined by using one-way analysis of variance [one-way ANOVA]. Differences with *P* < 0.05 were considered statistically significant.

## 3. Results

### 3.1. Effect of THHP on the Hepatic Pathological Changes in Different Animal Groups

**Figure 1:**
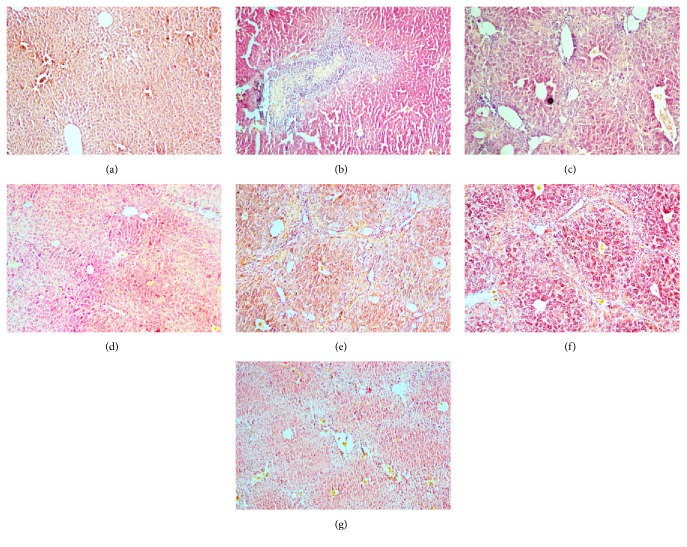
Effects of THHP on hepatic histopathological changes of CCl_4_-induced chronic liver disease mice. Hepar sections by Van Gieson (VG) staining at a magnification of ×100. (a) Blank; (b) CCl_4_; (c) CCl_4_ + colchicine (0.0001 g/kg); (d) CCl_4_ + THHP-H (5.53 g/kg); (e) CCl_4_ + THHP-M (2.67 g/kg); (f) CCl_4_ + THHP-L (1.33 g/kg); and (g) CCl_4_ + THSWD (8.50 g/kg).

To examine whether THHP could ameliorate the pathological changes in chronic liver disease model mice induced by CCl_4_, tissue sections were stained with VG for microscopy analysis.  Figure 1 revealed that hepatic tissue in the blank group had complete hepatic lobules, well-arranged hepatic cell cords, normal hepatic sinusoids, and no collagen fiber hyperplasia and inflammatory cell infiltrations. Hepatic tissue in the model group showed unclear structure of hepatic lobule, disrupted hepatic cell cords, some cellular swelling, fatty degeneration and necrosis, inflammatory cell infiltration, and fibrocyte, fibroblast, and collagen fiber in hepatic portal area, which had been ameliorated to some extent after treatment of THHP, THSWD, and colchicine (Figure 1). These results suggest that THHP inhibits CCl_4_-induced hepatic inflammation to relieve hepatic injury.

### 3.2. Effect of THHP on the Hepatic Function in Different Animal Groups

**Figure 2:**
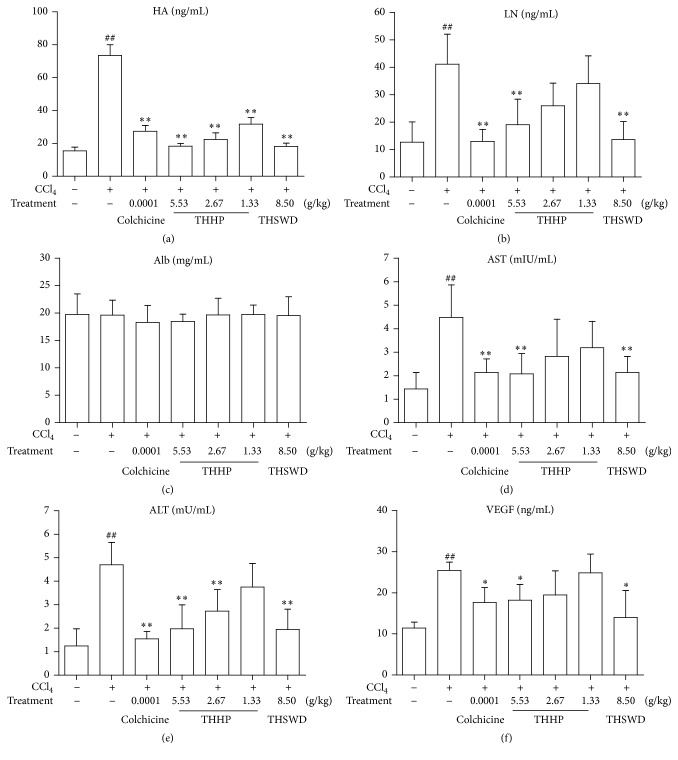
Effects of THHP on hepatic function, fibrosis correlated factors, and vascular endothelial growth factor (VEGF) of CCl_4_-induced chronic liver disease mice. (a) shows effect of THHP on serum hyaluronic acid (HA), (b) shows effect of THHP on serum laminin (LN), (c) shows effect of THHP on serum albumin (Alb), (d) shows effect of THHP on serum aspartate aminotransferase (AST), (e) shows effect of THHP on serum alanine aminotransferase (ALT), and (f) shows effect of THHP on hepatic VEGF. Data were represented as mean ± SD (*n* = 6–9 mice per group). ^##^ *P* < 0.01 compared with healthy controls; ^*∗*^ *P* < 0.05 and ^*∗∗*^ *P* < 0.01 compared with saline treated controls.

To examine if THHP could improve hepatic function of mice with CCl_4_-induced chronic liver disease, we measured serum aspartate aminotransferase, alanine aminotransferase, and albumin levels at the end of study. Colchicine was used as a positive control. As shown in Figure 2, after treatment with THHP at different concentrations, serum albumin level was not significantly changed (*P* > 0.05), but ALT and AST levels were significantly lower compared to the model group (*P* < 0.01). THHP decreased the concentrations of serum ALT in a dose-dependent manner. ALT and AST levels were also more reduced by THSWD as compared to the colchicine control though no statistical significance was observed. These results revealed that THHP has a role in inhibiting the hypohepatia of CCl_4_-induced chronic liver disease mice.

### 3.3. Effect of THHP on the Hepatic Fibrosis Correlated HA and LN in Different Animal Groups

To evaluate if THHP could inhibit hepatic fibrosis, we measured serum fibrosis correlated factors: hyaluronic acid and laminin. As shown in Figure 2, though HA level was slightly higher compared to the normal control group, the concentrations of serum HA in three THHP groups and THSWD group were significantly decreased after treatment as compared to the model group (*P* < 0.01). The reduction of serum LN in THHP-H and THSWD groups was also lower than that of colchicine group (*P* < 0.01). These results showed that THHP is capable of postponing the formation of hepatic fibrosis to some degree.

### 3.4. Effect of THHP on Microvessels Density in Different Animal Groups

**Figure 3:**
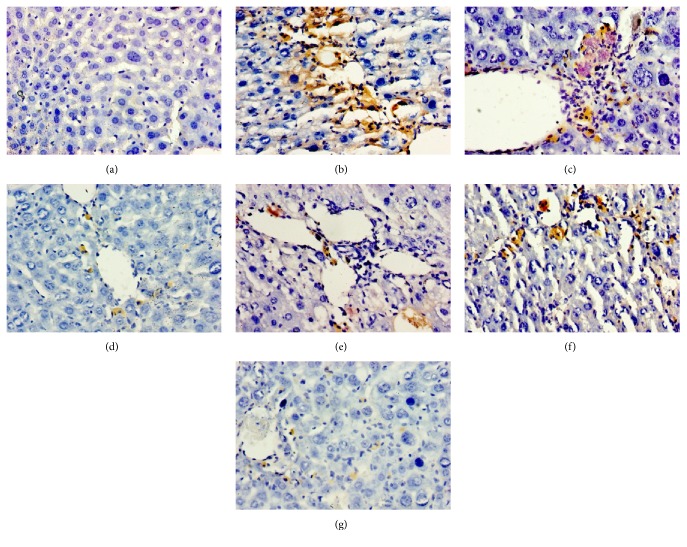
Effects of THHP on pathological microvessels in hepatic tissue of CCl_4_-induced chronic liver disease mice. Hepar sections by immunohistochemisty (IHC) staining at a magnification of ×200. (a) Blank; (b) CCl_4_; (c) CCl_4_ + colchicine (0.0001 g/kg); (d) CCl_4_ + THHP-H (5.53 g/kg); (e) CCl_4_ + THHP-M (2.67 g/kg); (f) CCl_4_ + THHP-L (1.33 g/kg); and (g) CCl_4_ + THSWD (8.50 g/kg).

**Figure 4:**
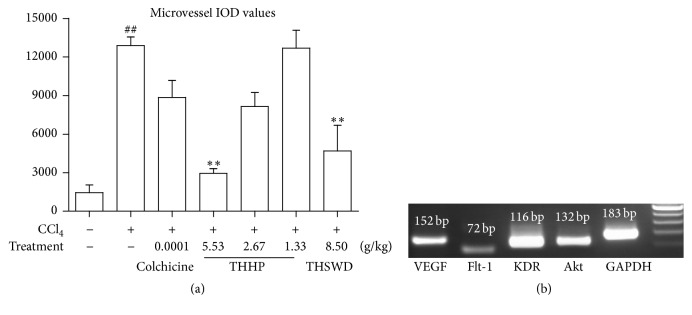
(a) Image analysis of integrated optical density (IOD) of pathological microvessels stained by immunohistochemistry in hepatic tissue of CCl_4_-induced chronic liver disease mice. Data were represented as mean ± SD (*n* = 6–8 mice per group). ^##^ *P* < 0.01 compared with healthy controls; ^*∗∗*^ *P* < 0.01 compared with saline treated controls. (b) Gel electrophotogram of target gene VEGF, Flt-1, KDR, and Akt amplification products of real-time fluorescent quantitation polymerase chain reaction.

To check if THHP could reduce hepatic pathological angiogenesis of CCl_4_-induced chronic liver disease mice, we measured the average hepatic microvessel density. As shown in Figures 3 and 4, after treatment, the vascular endothelial cells and microvessels stained by brown were evidently reduced, especially in THHP-H and THSWD groups; the microvessel integrated optical density (IOD) in THHP-H and THSWD groups was significantly decreased than that in the model group (*P* < 0.01). Furthermore, the effect of THHP in reducing pathological angiogenesis was better than that of colchicine group, but both IOD were higher compared to the normal control group. These results indicated that THHP has a certain effect on restraining hepatic angiogenesis of CCl_4_-induced chronic liver disease mice.

### 3.5. Effect of THHP on Serum VEGF Protein Expression in Different Animal Groups

Based on the results of THHP regulating pathological microvessels, we measured the content of VEGF protein in hepatic tissue. The ELISA analysis results showed that, after treatment with THHP and THSWD, though the level of VEGF protein in the four herbal medicine-treated groups has increased compared to that in normal group, compared with the model group, its level in THHP-H and THSWD groups has decreased significantly (*P* < 0.05) (Figure 2). These data showed that THHP is capable of reducing secretion of new vessel growth correlated factor VEGF after long term damage of CCl_4_-induced chronic liver disease mice.

### 3.6. Effect of THHP on mRNA Expression of VEGF, Flt-1, KDR, and Akt in Hepatic Tissues

**Figure 5:**
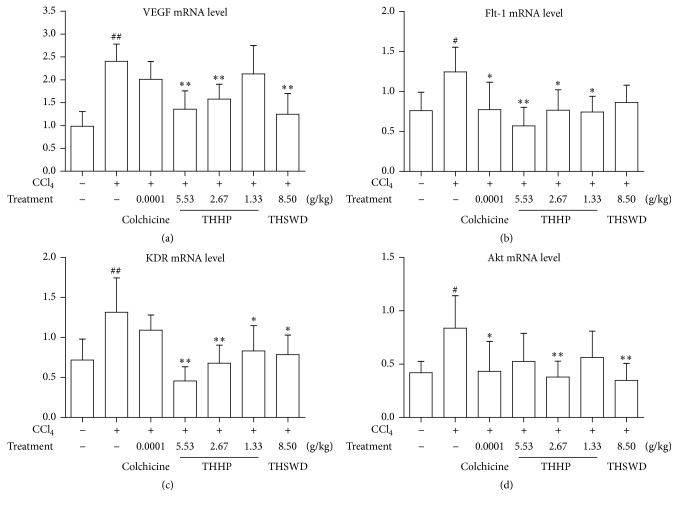
Effects of THHP on mRNA expression of VEGF, Flt-1, KDR, and Akt in hepatic tissue of CCl_4_-induced chronic liver disease mice. Data were represented as mean ± SD (*n* = 6–10 mice per group). ^#^ *P* < 0.05 and ^##^ *P* < 0.01 compared with healthy controls; ^*∗*^ *P* < 0.05 and ^*∗∗*^ *P* < 0.01 compared with saline treated controls.

In order to examine the mechanisms behind THHP's reduction of the pathological angiogenesis caused by CCl_4_, we further measured gene transcription of VEGF, Flt-1, KDR, and Akt in hepatic tissues. As shown in Figure 5, the quantitive PCR results showed that mRNA expressions of VEGF, Flt-1, and KDR were significantly reduced by THHP-H and THHP-M treatment (*P* < 0.01, *P* < 0.05). Akt mRNA expression was reduced by THHP-M and THSWD treatment (*P* < 0.01). And the efficacy of THHP was superior to colchicine treatment. Our data revealed that THHP may exert its effect though lowering the transcriptions of genes involved in pathological angiogenesis.

### 3.7. Effect of THHP on Protein Expression of Akt, pAkt, Flt-1, and KDR in Hepatic Tissues

**Figure 6:**
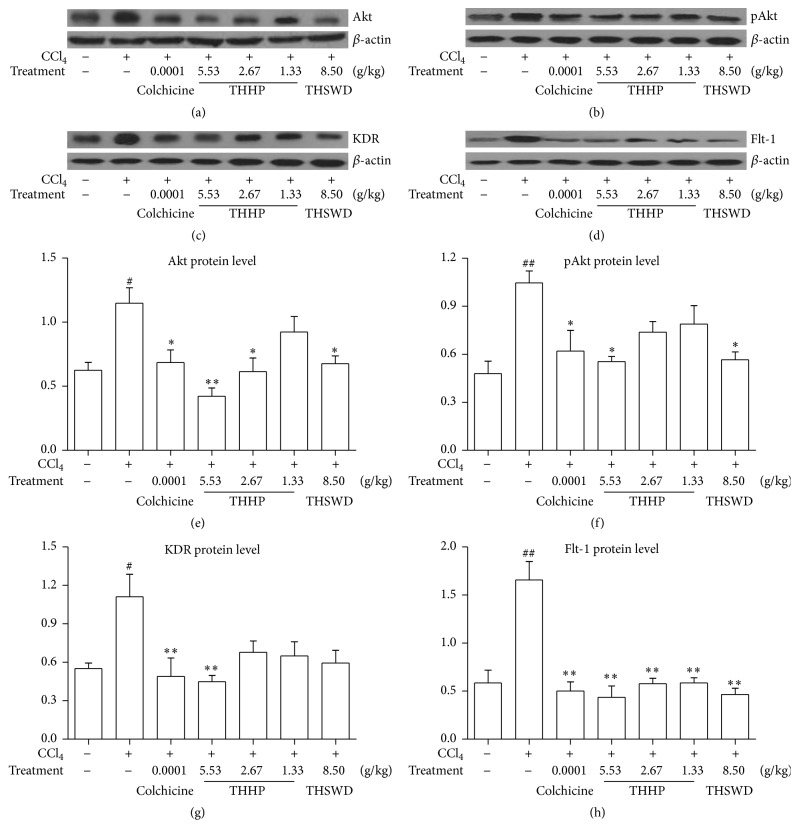
From (a) to (d), the western blot band pictures of Akt, pAkt, KDR, and Flt-1 in each group; from (e) to (h), effects of THHP on protein expression of Akt, pAkt, KDR, and Flt-1 in hepatic tissue of CCl_4_-induced chronic liver disease mice. Data were represented as mean ± SD (*n* = 6–8 mice per group). ^#^ *P* < 0.05 and ^##^ *P* < 0.01 compared with healthy controls; ^*∗*^ *P* < 0.05 and ^*∗∗*^ *P* < 0.01 compared with saline treated controls.

As expected, in Figure 6, the western blot results indicated that THHP could reduce the protein expression of Akt, pAkt, Flt-1, and KDR in hepatic tissue of CCl_4_-induced chronic liver disease mice at the dose of 5.53 g/kg (*P* < 0.05, *P* < 0.01). We also found that the protein expression of Akt, pAkt, and Flt-1 in hepatic tissue could be decreased by THSWD (*P* < 0.05, *P* < 0.01). Combining the above results of ELISA and PCR, we considered that blockage of angiogenesis-associated VEGF signaling via inhibiting the expression of Akt and pAkt and VEGF and its receptors (KDR and Flt-1) to attenuate its signal activation is one of the possible mechanisms of THHP treating CCl_4_-induced chronic liver disease.

## 4. Discussion

Chronic liver disease characterized by hepatic inflammation and fibrosis is caused by various factors. Its formation mechanisms are complicated. In chronic liver diseases, angiogenesis, the formation of new hepatic pathological microvessels from preexisting ones, may contribute to progressive hepatic fibrosis and the development of hepatocellular carcinoma [14, 15]. In recent years, experts also have found that pathological angiogenesis is presented in hepatic inflammation processes [16]. But tissue pathological angiogenesis and posttrauma blood capillary repair are two different pathophysiological processes. Most of pathological neovessels in chronic liver disease are more immature and are the useless blood vessels and no good for getting better of chronic liver disease. The activation of hepatic stellate cell (HSC) and its transformation into myofibroblast are the center link of hepatic fibrosis formation, and activated HSCs express proangiogenic factors, which promote proliferation and migration of hepatic sinusoidal endothelial cells and in turn accelerate fibrosis [17]. Research indicated that VEGF level in chronic hepatitis, hepatic cirrhosis, and primary liver cancer was significantly higher and related to the degree of hepatic fibrosis; pathological angiogenesis mainly located in fiber interval of portal area periphery and had the tendency of invading from portal area periphery to parenchyma in accordance with fibrosis development [16, 18]. Chronic liver disease or (with) liver fibrogenesis is linked to VEGF induced angiogenesis [8, 19]. Because the useless immature neovessels in hepatic fibrosis area could cause the formation of vascular plexus and split around the regenerating hepatocytes and hinder the exchange between sinusoid and hepatocyte, the regenerating hepatocyte could not construct the normal portal vein branch, which could aggravate the hepatic cell injury [20]. Therefore, hepatic fibrosis and hepatic pathological angiogenesis form viscous cycle: an increase of pathological angiogenesis will cause more fibrotic growth. This suggests that controlling pathological angiogenesis may be beneficial for inversion of hepatic fibrosis.

VEGF has high affinity for two receptors: KDR and Flt-1. Flt-1 mediates the cytoskeleton rearrangement to migrate; KDR mainly mediates the endothelial hyperplasia maintaining the cell survival. KDR exerts the major role in VEGF induced angiogenesis and vascular permeability [21]. KDR antibody can singlehandedly inhibit the pathological angiogenesis induced by VEGF through inhibition of the KDR downstream tyrosine kinase signal transduction system [22]. In receptor tyrosine kinase signal transduction system, the survival of vascular endothelial cell after proliferation and differentiation mainly depends on PI3K-Akt that is an important transduction pathway of survival signal; it participates in the angiogenesis-associated signaling pathway like VEGF-mediated endothelium signal transduction: PI3K and upstream VEGFR-2 (KDR) form compound and activate, generate second messenger, activate phosphoinositide dependent protein kinase (PDK), and activate the downstream serine-threonine protein kinase (Akt), and then generate the effect of vascular endothelial cell activation, so the level of activated Akt [which also is phosphorylated Akt (pAkt)] is the important index that represents the activity of PI3K-Akt [23]. So if this pathway is interrupted, the endothelium activation will be inhibited and pathological angiogenesis will be reduced.

At present, TCMs antagonizing chronic liver diseases with hepatic fibrogenesis focus more on anti-inflammatory, inhibiting lipid peroxidation and extracellular matrix synthesis, promoting extracellular matrix degradation, restraining platelet-derived growth factor, inducing HSC apoptosis, and inhibiting collagen synthesis [24]. For example, Shen-Qi-Nei-Jin showed antifibrotic effects on hepatic fibrosis rats induced by CCl_4_ through inhibiting HSCs activation and inducing apoptosis of HSCs [25]. And the modified Si-Ni relieved liver injury in rats by the inhibition of the inflammatory reaction and apoptosis and the promotion of liver tissue regeneration [26]. The studies about single* Semen Persicae* (Taoren) or* Flos Carthami* (Honghua) are mostly on cardiovascular pharmacology like antagonizing thrombus, inhibiting inflammation, analgesia, and ameliorating microcirculation [27, 28]. The researches with TCMs treating chronic liver disease from intervening pathological angiogenesis are few.

Meanwhile, like effects of ameliorating dimethylnitrosamine-induced liver injury and fibrosis in rats through downregulation of antioxidant enzymes and inducing oxidative modification were testified with* Radix Scutellariae Baicalensis* (Huangqin) and* Radix et Rhizoma Rhei Palmati* (Dahuang) [29], and antioxidative action of THHP was not mentioned in present research; additional action and mechanism studies are still necessary to carry out. Therefore, based on the previous researches, our studies tried to develop TCMs other pharmacologic actions with THHP.

THHP is composed of the two chief medicinals of THSWD and the effect is more focused on promoting blood circulation than that of the latter. Our findings revealed that specific concentration of THHP alleviated hepatic injury and inflammation and decreased hepatic pathological angiogenesis (which were slightly better than that of colchicine from the point of the pathology) and improved hepatic function to certain degree and also testified that THHP had certain effects on downregulating VEGF and its main receptors and could inhibit both mRNA and protein expression of Akt and protein expression of pAkt to inhibit the pathological angiogenesis to protect hepar. Those findings suggest that angiogenesis-associated VEGF and Akt and its upstream or downstream activating factors may be as the targets of THHP for anti-inflammation and antifibrosis.

Due to* Semen Persicae* (Taoren) extract has been reported to attenuate hepatic collagen fibers of experimental hepatic fibrosis rats [30]; and* Flos Carthami* (Honghua) active constituent safflower yellow or hydroxy safflower yellow-A (HSYA) has also been reported to improve hepatic function and relieve hepatitis and hepatic fibrosis [31, 32]; in THHP,* Semen Persicae* (Taoren) and* Flos Carthami* (Honghua) are much possible to play a synergistic effect on chronic liver disease model mice derived from each herbal medicine's components.

From our results, reducing pathological angiogenesis by inhibiting expression of VEGF and Akt is an important effect of THHP. But recent research showed that VEGF was also required for hepatic tissue repair and fibrosis resolution although in a mouse model of liver fibrosis resolution VEGF promoted fibrogenesis [33]. Combining the aforementioned, pathological microvessels formed in early phase are usually nonfunctioning, inhibit angiogenesis during the early stage, and may be beneficial to reduce hepatic fibrogenesis. So, future researches should focus on the effectiveness and mechanism of THHP treating the early stage of chronic liver disease.

There are several limitations to the present study. First, differences of therapeutic effectiveness between THHP and THSWD remain unclear. Second, although THHP showed the multiple effects of treating CCl_4_-induced chronic liver disease, whether the mechanism of THHP decreasing pathological angiogenesis is responsible for the relief of hepatic inflammation and fibrosis also remains to be clarified. Third, our experimental results of THHP still need to be further confirmed in human trials. Finally, due to complex combination of components within THHP, the constituents that exert the dominant efficacy will be left for future work.

## 5. Conclusion

In summary, we demonstrated that THHP has a notable effect on decreasing hepatic pathological angiogenesis and positive therapeutic effectiveness in chronic liver disease with hepatitis and fibrosis* in vivo*. The mechanism of action may lie in inhibiting the VEGF and its upstream and downstream signaling pathway to reduce the pathological angiogenesis, which provides a promising therapeutic alternative for clinical patients.
